# Supplementary material for: Decoding the Role of Melatonin Structure on Plasmodium falciparum Human Malaria Parasites Synchronization Using 2-Sulfenylindoles Derivatives
Source: Biomolecules. 2022 Apr 26;12(5):638. doi: 10.3390/biom12050638 (PMC9138683; doi:10.3390/biom12050638)
Supplement: Supplementary file 1 [file biomolecules-12-00638-s001.zip › biomolecules-1649074-supplementary.pdf]

Supplementary Table S1 – Oligonucleotides used in real-time PCR experiment for melatonin treatment.

**GENES**	**Primer Name**	**Oligonucleotides (5′ → 3′)**
CDPK1 PF3D7_0217500	CDPK1-R	GGGTTTATCGCTAGGTCCTCC
	CDPK1-F	GGGTGTTCACAAAGTTCAAACGT
CDPK2 PF3D7_0610600	CDPK2-R	ACACCACTAGACCAGATATCACATT
	CDPK2-F	GGAACAGGAGAATTTACAACGACT
CDPK3 PF3D7_0310100	CDPK3-R	CCCCGTCATTGTCTACGTCA
	CDPK3-F	GTGATGGCAGTGGGAAAATTGA
CDPK4 PF3D7_0717500	CDPK4-R	TGTATATTTCCCCGCTTCCACT
	CDPK4-F	CATACTTCTCTCAGGGTGCCC
CDPK5 PF3D7_1337800	CDPK5-R	TCGGGTGCTATATAATAAGGTGTCC
	CDPK5-F	TGATTGGGGATTCGCTAGTAAATGT
CDPK6 PF3D7_1122800	CDPK6-R	TCCAAAGCCTGAAGAATCCGA
	CDPK6-F	ACGGATCATTGAGTCATCGAGA
CDPK7 PF3D7_1123100	CDPK7-R	ACCTTGCCCTAACTGTTCATGT
	CDPK7-F	AGGGATGAATGGGTACAAGCG
GCα PF3D7_1138400g	GCα-R	ACCGACCACACCTGCAATAA
	GCα-F	ACTCTTTCAATAACGACAAAGCAGA
GCβ PF3D7_1360500	GCβ -R	TCTCCAGGTATCCCATTTGATTCA
	GCβ-F	ATGGCTCATGTGTTGGTGGT
PfPKG PF3D7_1436600	PKG-R	TCTTCTGCATAAGTTTCACT
	PKG-F	TCAAAGACATAAAGGATCAC
PfPDEα PF3D7_1209500	PDEα-R	TCTTCGTTAATTTTGAGTGT
	PDEα-F	TGGCAAATATTCTGTCTTAT
PfPDEβ PF3D7_1321500	PDEβ-R	AAACTTCATTGTGTTTTGTC
	PDEβ-F	GTTGGTGTCAGAGAGTTTTA
PfPDEγ PF3D7_1321600	PDEγ-R	CTACCAAAATGTCCTATATCAT
	PDEγ-F	AAGTAATTTAAATACAGCGAAT
PfPDEδ PF3D7_1470500	PDEδ-R	ATAAACTTCCTCATGTTTGTAT
	PDEδ-F	AACATTTTCCATATTACAACTT

Supplementary Table S2 – Effects of 2-sulfenylindoles on the parasitemia of P. falciparum after 48 h of incubation with 500 nM compounds in combination with 100 nM melatonin.

ID	Parasitemia (%)	
	Treatment with compound (500nM)	Treatment with compound (500nM) + Melatonin (100nM)
**(3)**	98.22 ± 2.28 %	129.8 ± 6.78 %
**(7)**	96.55 ± 2.39 %	122.9 ± 5.60 %
**(8)**	101.6 ± 1.71 %	133.5 ± 8.06 %
**(10)**	100.6 ± 2.14 %	130.2 ± 6.02 %
**(14)**	97.75 ± 3.41 %	128.5 ± 4.13 %
**(16)**	111.8 ± 1.76 %	127.4 ± 4.10 %
**(17)**	98.57 ± 2.95 %	123.6 ± 3.11 %
**(18)**	97.99 ± 2.61 %	113.9 ± 2.90 %
**(19)**	100.5 ± 1.81 %	118.5 ± 2.85 %
**(20)**	109.7 ± 4.07 %	122.3 ± 3.71 %
**(21)**	104.4 ± 1.00 %	111.9 ± 2.78 %
**(25)**	107.2 ± 2.46 %	112.8 ± 2.93 %
**(26)**	92.66 ± 4.17 %	110.4 ± 3.18 %
**(28)**	109.3 ± 1.81 %	115.4 ± 2.98 %
**Melatonin**	107.5 ± 4.32 %	113.5 ± 2.75 %
**Control**	100.6 ± 1.88 %	100.7 ± 2.65 %

Data are presented as the percentage of parasitemia normalized to that of the control group treated with solvent (DMSO). Experiments were performed 3 independent times in triplicate.

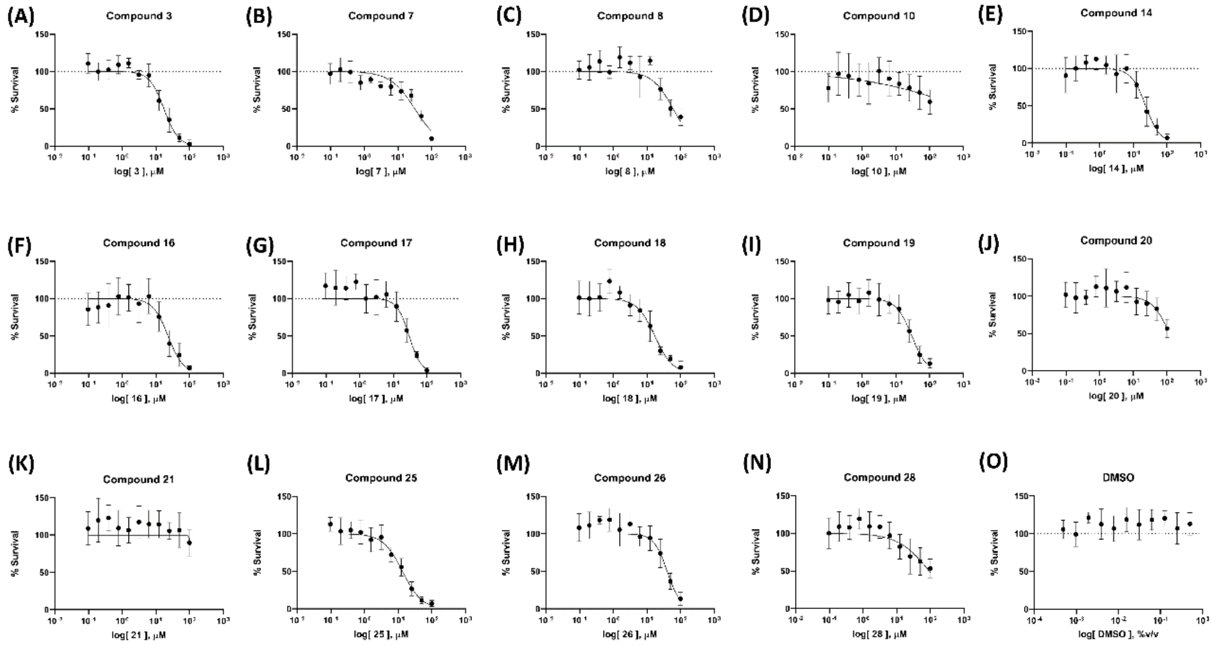

Figure S1 - Effect of indole derivatives in HEK293 cells. Cells were treated with concentrations ranging from 0 to 100 μM for compounds 3(A), 7(B), 8(C), 10(D), 14(E), 16(F), 17(G), 18(H), 19(I), 20(J), 21(K), 25(L), 26(M), 28(N). Each point represents mean ±SD for four independent experiments.

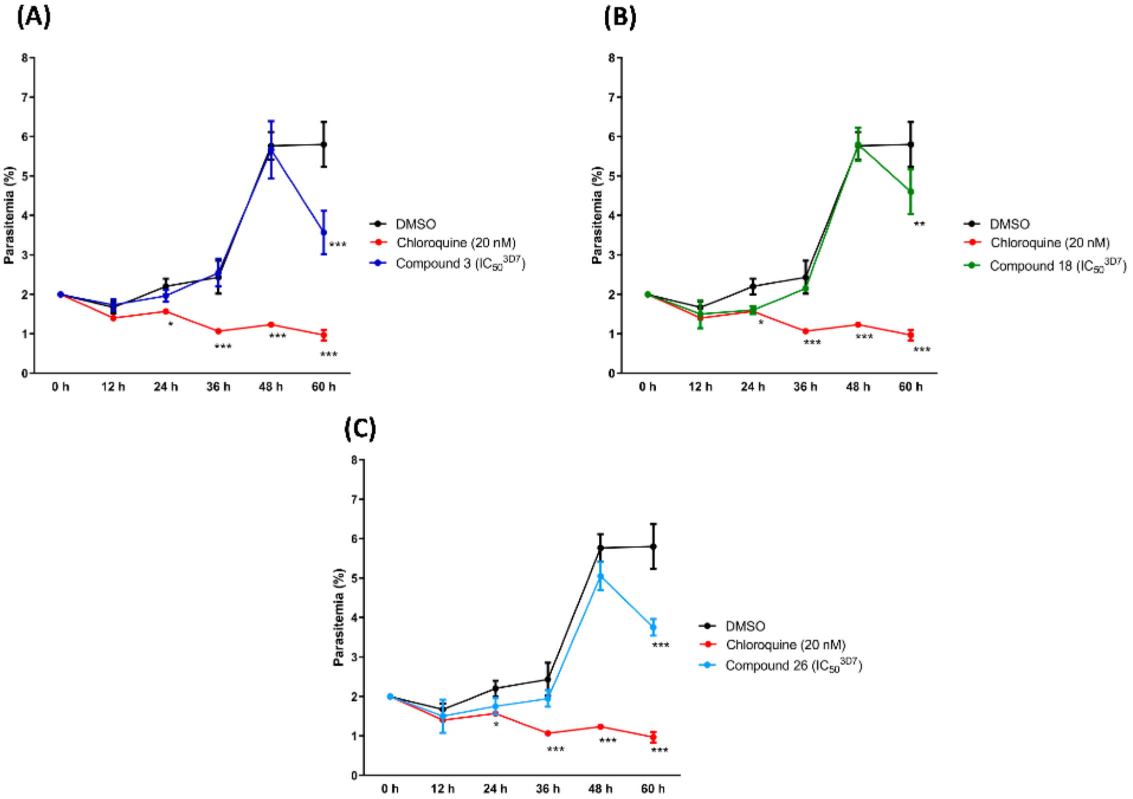

Figure S2 – Parasitemia reduction obtained after incubation with compound **3** (**A**), compound 18 (**B**), or compound **26** (**C**). Data are means ± SD. Parasitemia determination was performed every 12 hours after drug administration to determine the stage activity. Parasites synchronized to the ring stage were treated with the compounds (**3**, **18**, and **26**), with the best activity against 3D7 parasites. The chosen treatment concentration was the IC_{50} values obtained for these compounds against the 3D7 strain.
